# ﻿Changes in metabolic overweight phenotypes over time and risk of nephrolithiasis: a cohort study

**DOI:** 10.1186/s12889-024-19229-8

**Published:** 2024-07-16

**Authors:** Yang Cheng, Hui Zheng, Hongli Yin, Donghua Yin, Hui Wang, Ying Wang, Qiang Tang, Shouyong Gu

**Affiliations:** 1 Center for Health Management, Jiangsu Province Geriatric Hospital, 2 Yi-He Road, Nanjing, 210009 China; 2 School of Sports and Health, Nanjing Sport Institute, 8 Ling-Gu Temple Road, Nanjing, 210014 China; 3 Jiangsu Collaborative Innovation Center for Sport and Health Project, Nanjing, China; 4 Institute of Geriatric Medicine, Jiangsu Province Geriatric Hospital, 65 Jiang-Su Road, Nanjing, 210009 China

**Keywords:** Nephrolithiasis, Metabolic abnormalities, Overweight, Prospective cohort study

## Abstract

**Background:**

Overweight/obesity is considered an independent risk factor for nephrolithiasis, but little is known about its effect on nephrolithiasis according to metabolic health status.

**Objectives:**

We aimed to investigate the association between various metabolic overweight phenotypes and the occurrence of nephrolithiasis. It also explores whether changes in these phenotypes over time influence the risk of nephrolithiasis.

**Materials and methods:**

A total of 10,315 participants free of nephrolithiasis who underwent an annual health checkup from 2017 to 2022 were included in our prospective cohort study. They were categorized into four groups according to the presence of overweight and metabolic abnormalities (MA). The primary endpoint was the occurrence of renal stones. Multivariable Cox analysis was conducted to elucidate the relationship between metabolic overweight phenotypes and incident nephrolithiasis.

**Results:**

During a median follow-up duration of 4.02 years, nephrolithiasis occurred in 1,468 (14.23%) participants. In the full cohort, we observed that the 5-year cumulative incidences of nephrolithiasis were highest in the metabolically healthy overweight (MHO) and metabolically abnormal overweight (MAO) groups. The hazard ratios (HRs) for nephrolithiasis, relative to metabolically healthy normal weight (MHNW), ranged from 1.19 (95% CI:1.03–1.37; MHO) to 1.32 (95% CI:1.15–1.51; MAO). Furthermore, individuals with persistent MHO throughout follow-up were at a 1.42-fold increased risk of nephrolithiasis (P < 0.001), and 32.17% of individuals experienced changes in phenotype during follow-up. Among MAO subjects, those who transitioned to MHO and MHNW had a 26% and 45% lower risk of incident nephrolithiasis, respectively, compared to those who persisted in the MAO phenotype.

**Conclusion:**

Individuals in the MHO and MAO groups exhibit an elevated risk of incident nephrolithiasis in this prospective cohort study. A significant proportion of nephrolithiasis cases may be potentially preventable through the appropriate management of metabolic risk factors for MAO subjects.

**Supplementary Information:**

The online version contains supplementary material available at 10.1186/s12889-024-19229-8.

## Introduction

Nephrolithiasis, commonly known as kidney stones, refers to the formation of mineral deposits within the kidney's collecting system, such as the renal calyx and renal pelvis [1]. The prevalence and incidence of nephrolithiasis have been on a rise globally, bringing huge health and economic burdens [2–4]. While the onset of nephrolithiasis is influenced by age, gender and ethnicity [5], its underlying pathogenesis remains incompletely elucidated.

Epidemiologic studies have indicated that higher body mass index (BMI) is associated with elevated risk of symptomatic kidney stones [6, 7]. Yet, the health implications of overweight extend beyond BMI, encompassing aspects of fat distribution and lipid metabolism [8–10]. Of note, metabolic abnormalities (MA) also play a vital role in the initiation and recurrence of kidney stones [11]. Thus, more attention needs to be paid to different status of metabolic abnormalities versus overweight and explore their relationships with kidney stones.

Many overweight or obese individuals are diagnosed with MA [12], but a large percentage of overweight or obese people remain metabolically healthy and are identified as having a metabolically healthy overweight (MHO) phenotype [13]. The question lingers as to whether this distinct MHO phenotype serves as a predisposing factor for kidney stone formation [14]. Hence, this large-scale prospective cohort study aimed to shed further light on the relationship between different metabolic overweight phenotypes and incident nephrolithiasis. Additionally, we examine the changes of different metabolic overweight phenotypes in individuals during follow-up and further explored whether such phenotypic shifts over time may influence the new development of incident nephrolithiasis.

## Methods

### Study design and participants

All subjects were recruited from the health management institution of Jiangsu Province Geriatric Hospital (Nanjing, China). Initially, a total of 26,621 subjects who had undergone a health examination and voluntarily provided informed consent were recruited in 2017. Serial follow-up medical examinations were conducted at the center until 2022. Among these participants, we excluded samples with nephrolithiasis at baseline; those with missing data on BMI, systolic blood pressure, diastolic blood pressure, blood glucose, total cholesterol (TC), triglyceride (TG), high-density lipoprotein-cholesterol (HDL-C) and low-density lipoprotein-cholesterol (LDL-C); and those with $\le$ 1 follow-up data were excluded. Finally, we included 10,315 subjects in our study. The Institutional Review Board of Jiangsu Province Geriatric Hospital approved this study.

### Anthropometric and physiological measurements

According to the standard procedures, we measured the height, weight and blood pressure (BP) as previously reported [15]. BMI was calculated as weight (kg) / the square of height (m^2^). All participants underwent abdominal ultrasonography (US) at baseline and each visit. Abdominal US was performed by experienced radiologists in accordance with standards set forth by the Chinese Ministry of Health. Images were captured, when subjects were in the supine position with the right arm raised above their head. Nephrolithiasis was diagnosed by the presence of hyperechoic structures that caused acoustic shadowing in the collecting system on US.

### Biochemistry detection

After overnight fasting of 8 h, five milliliters of venous blood were drawn by a research nurse for detecting plasma concentration of fasting blood glucose (FBG) and lipid profile parameters: TC, TG, HDL-C and LDL-C.

### Definition of metabolic overweight phenotypes

1. Metabolic syndrome: According to the “Chinese Guidelines for the Prevention and Treatment of Type 2 Diabetes (2020 Edition)”, those with or exceeding the following two components are defined as MA.
  a. Hyperglycemia: FBG ≥ 6.1 mmol/L and (or) diagnosed with diabetes and treated;
  b. Hypertension: blood pressure ≥ 130/85 mmHg and (or) confirmed hypertension and treated;
  c. TG ≥ 1.70 mmol/L;
  d. HDL-C < 1.04 mmol/L in men and < 1.29 mmol/L in women.
2. Overweight phenotype: Based on the China Obesity Working Group, a BMI value ≥ 24 kg/m^2^ is determined as overweight [16].

Based to the status of MA and overweight, the subjects were categorized into four phenotypes: metabolically healthy normal weight group (MHNW); MHO; metabolically abnormal normal weight group (MANW); metabolically abnormal overweight group (MAO).

### Data collection of other characteristics

The trained physicians collected comprehensive information, including demographic characteristics (age and sex) and medical history (hypertension and diabetes) through face-to-face interviews.

### Statistical methods

﻿Continuous variables were presented as mean $\pm$ standard deviation (*x̅* $\pm$ *S*) for normally distributed data, or median with interquartile range for non-normally distributed data. Categorical variables were expressed as frequencies and percentages (n, %). Analysis of variance and the chi-square test were used to compare the classification data of different groups as appropriate.

The follow-up period was calculated from baseline until the date of incident nephrolithiasis or last visit, whichever occurred first. Rates of nephrolithiasis were defined as the crude incidence rates, indicating the number of outcomes per 1000 person-years at risk. Cumulative incidence was estimated using the Kaplan–Meier method (1- Kaplan–Meier estimate). Cox regression was utilized to calculate hazard ratios (HRs) and 95% confidence intervals (CIs) of nephrolithiasis events among different groups. Different models were constructed as follows: model 1 represents a crude risk without adjustment; model 2 was adjusted for age and sex.

To test the robustness of our findings, we performed the following sensitivity analyses: (1) in 6,997 subjects who remained the same phenotype without transition to other phenotypes at every visit; (2) with exclusion of individuals who developed renal stones during the first year. All data analyses were conducted in R version 4.1.1 (R Foundation for Statistical Computing), with a *P* value of < 0.05 being considered as statistically significant.

## Results

### Baseline characteristics of the participants

**Fig. 1:**
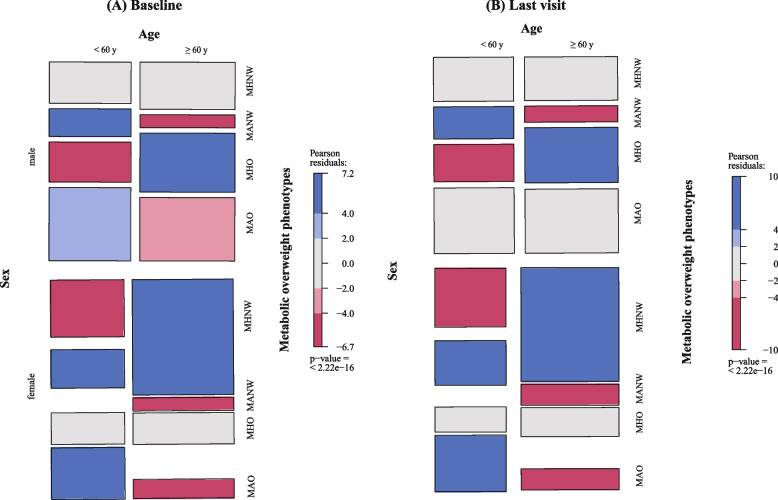
The prevalence of the four different metabolic overweight phenotypes by age and sex. **A** Baseline and **B** the last follow-up. MHNW, metabolically healthy normal weight group; MHO, metabolically healthy overweight group; MANW, metabolically abnormal normal weight group; MAO, metabolically abnormal overweight group

Table 1 presents comparisons of the clinical profiles between participants with metabolic overweight phenotypes in this longitudinal study. Of the 10,315 participants, 5,530 (53.61%) participants were overweight, and 4,305 (41.74%) exhibited metabolic abnormalities. The prevalences of MHNW, MHO, MANW, and MAO were 34.58%, 23.68%, 11.81% and 29.93%, respectively. Among the normal weight individuals, MANW were characterized by higher proportions of male, hypertensive and diabetic subjects compared to MHNW group. Among the overweight subjects, there were more females and younger people in the MHO group than in the MAO group. We also showed the comparisons of the clinical profiles between participants with and without nephrolithiasis in Supplementary Table 1. Subjects with nephrolithiasis were more likely to be older, had higher systolic blood pressure and diastolic blood pressure, higher levels of FBG, TC, TG, LDL, higher prevalence of hypertension and diabetes compared to those without nephrolithiasis. In addition, for male, the prevalence of MHO was higher in older than in young people at baseline and the last visit, while the difference was not significant for female (Fig. 1).

**Table 1:** Baseline characteristics of participants among 4 baseline phenotypes classified by the presence obesity and/or metabolic abnormality

**Variables **^b^	**Overall**	**Normal weight**		**Over-weight**		*P* value ^a^
		**MHNW**	**MANW**	**MHO**	**MAO**	
**Participants (n, %)**	10,315	3,567(34.58%)	1,218(11.81%)	2,443(23.68%)	3,087(29.93%)	-
**Follow-up duration (years)**	4.02(1.99, 5.01)	4.02(2.01,5.00)	4.12(1.98–5.02)	4.01(1.98–4.99)	4.04(1.97–5.01)	0.004
**Age**	57(46,67)	52 (38, 63)	65 (55, 75)	55 (44, 65)	61 (52, 70)	< 0.001
**Male (n, %)**	6326(61.33%)	1541 (43.2%)	687 (56.4%)	1742 (71.3%)	2356 (76.3%)	< 0.001
**BMI (kg/m**^**2**^**)**	24.20(22.10–26.40)	21.7 (20.3, 22.9)	22.6 (21.5, 23.3)	25.8 (24.8, 27.2)	26.6 (25.2, 28.1)	< 0.001
**Systolic pressure (mmHg)**	130(118,145)	119 (110, 130)	140 (130, 152)	127 (118, 140)	140 (130, 152)	< 0.001
**Diastolic pressure (mmHg)**	77(70,85)	72 (66, 79)	79 (72, 86)	77 (71, 84)	83 (76, 90)	< 0.001
**Fasting blood glucose (mmol/L)**	5.59(5.21,6.11)	5.33 (5.03, 5.66)	6.16 (5.55, 6.85)	5.47 (5.15, 5.79)	6.14 (5.54, 6.87)	< 0.001
**Total cholesterol (mmol/L)**	4.99(4.37,5.64)	4.95 (4.34, 5.55)	5.10 (4.44, 5.81)	4.98 (4.42, 5.6)	5.01 (4.37, 5.74)	< 0.001
**Triglyceride (mmol/L)**	1.29(0.93,1.82)	0.97 (0.75, 1.25)	1.74 (1.16, 2.22)	1.19 (0.94, 1.47)	1.93 (1.44, 2.56)	< 0.001
**HDL-C (mmol/L)**	1.32(1.13,1.56)	1.53 (1.32, 1.75)	1.24 (1.07, 1.46)	1.34 (1.18, 1.51)	1.13 (1, 1.29)	< 0.001
**LDL-C (mmol/L)**	3.03(2.49,3.61)	2.94 (2.42, 3.49)	3.05 (2.43, 3.67)	3.14 (2.65, 3.68)	3.05 (2.46, 3.68)	< 0.001
**Hypertension (n, %)**						< 0.001
**No**	4347(42.14%)	2516 (70.5%)	176 (14.5%)	1282 (52.5%)	373 (12.1%)	
**Yes**	5968(57.86%)	1051 (29.5%)	1042 (85.6%)	1161 (47.5%)	2714 (87.9%)	
**Diabetes (n, %)**						< 0.001
**No**	7612(73.80%)	3383 (94.8%)	518 (42.5%)	2289 (93.7%)	1422 (46.1%)	
**Yes**	2703(26.20%)	184 (5.2%)	700 (57.5%)	154 (6.3%)	1665 (53.9%)	
**Nephrolithiasis (n, %)**						< 0.001
**No**	8847(85.77%)	3159 (88.6%)	1062 (87.2%)	2077 (85.0%)	2549 (82.6%)	
**Yes**	1468(14.23%)	408 (11.4%)	156 (12.8%)	366 (15.0%)	538 (17.4%)	

^a^Comparisons between groups analyzed by ANOVA or Kruskal–Wallis test for continuous variables; and Chi-squared test was used to examine the differences for categorical variables

^b^*MHNW M*etabolically healthy normal weight, *MHO *Metabolically healthy overweight, *MANW* Metabolically abnormal normal weight, *MAO* Metabolically abnormal overweight, *BMI* Body mass index, *HDL-C* High density lipoprotein cholesterol, *LDL-C *Low-density lipoprotein cholesterol

### Cumulative incidence of nephrolithiasis in metabolic overweight phenotypes

**Fig. 2:**
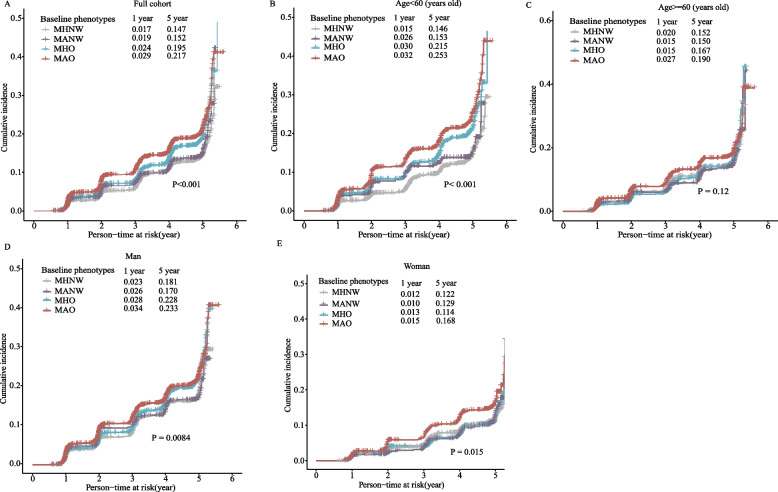
Cumulative incidence of nephrolithiasis at different metabolic overweight phenotypes. **A** in full cohort; **B** in younger age group; **C** in older age group; **D** in man group; **E** in woman group

We examined the cumulative incidence of nephrolithiasis in the four phenotypes over a 5-year study period. In the full cohort, the overall five-year cumulative incidences in the MAO and MHO groups were 21.7% and 19.5%, respectively, which were higher than the incidences in the MHNW and MANW groups (MHNW: 14.7%; MANW:15.2%) (Fig. 2A). When stratified by age (< 60 years old vs. $\ge$ 60 years old) and gender, similar trends were identified among different metabolic overweight phenotypes in younger age group and males, producing statistically significant differences (*P* < 0.05, Fig. 2B-E).

### Association between the four metabolic overweight phenotypes and the risk of nephrolithiasis

During a median follow-up duration of 4.02 years (interquartile range, 1.99–5.01 years), 1,468 participants developed nephrolithiasis (incidence rate, 3.98 cases per 1,000 person-years). Compared with normal weight group, overweight group had an increased risk of incident nephrolithiasis (HR, 1.25; 95% CI: 1.12–1.39). Additionally, individuals with MA were at a 1.14-fold increased risk (95% CI: 1.03–1.27) of incident nephrolithiasis compared to individuals without MA (Supplementary Table 2). The crude incidence rates per 1,000 patient-years of nephrolithiasis were 3.18, 3.50, 4.25 and 4.87 in the MHNW, MANW, MHO and MAO groups, respectively. As expected, compared with the MHNW group, overweight subjects in the MHO (HR, 1.19; 95% CI, 1.03–1.37) and MAO (HR, 1.32; 95% CI, 1.15–1.51) groups were at an increased risk of incident nephrolithiasis (Table 2). Subgroup analyses according to age and gender, the finding was consistent only in younger man. In women < 60 years old, a significantly increased risk of kidney stones in MAO alone was identified, suggesting the need for a combination of metabolic abnormalities and excess weight among younger women (Supplementary Table 3 and Figure S1).

**Table 2:** Hazard ratios for kidney stone according to presence of obesity and metabolic abnormality

Baseline phenotypes (*n* = 10,315)		MHNW	MANW	MHO	MAO
Patient-years		12,806	4,463	8,614	11,037
Incidence rate per 1,000 patients-years (number of cases)		3.18(408)	3.50(156)	4.25(366)	4.87(538)
Crude HR^a^	HR (95% CI)	1.00(reference)	1.10(0.91–1.32)	1.36(1.18–1.57)	1.54(1.35–1.75)
	*P*-value		0.328	< 0.001	< 0.001
Adjusted HR^b^	HR (95% CI)	1.00(reference)	1.03(0.86–1.25)	1.19(1.03–1.37)	1.32(1.15–1.51)
	*P*-value		0.725	0.020	< 0.001

^a^A crude analysis without adjustment

^b^Adjusted for age, sex

### Sensitivity analyses

We found the consistent results as above in the sensitivity analyses. First, we analyzed 6,997 subjects who had not converted to other phenotypes during follow-up. The distinct features of the four baseline phenotypes were also found among the four persistent phenotypes as described in Supplementary Table 4. In multivariable-adjusted model, compared with persistent MHNW controls, persistent MAO individuals were at a 1.42-fold (95% CI, 1.21–1.66) increased risk of nephrolithiasis (Supplementary Table 5). In addition, sensitivity analyses with excluding nephrolithiasis occurred in the first year, also showed the same results (Supplementary Table 6).

### Changes in metabolic overweight phenotypes during follow-up

The status of overweight and MA can change over time, therefore, we measured how many baseline phenotypes changed to other phenotypes during the follow-up (Supplementary Table 7). The average follow-up visits for the participants were 3.83 $\pm$ 1.40 times and 32.17% of the subjects experienced phenotype changes during this period. Approximately 50–80% of participants retained their baseline phenotypes at the last visit. It is worth noting that during the follow-up period, 69.55% of MAO individuals showed no phenotypic changes, while 10.46% and 16.46% of the subjects changed to the MANW and MHO phenotypes, respectively.

### Risk of incident nephrolithiasis caused by metabolic overweight phenotypes transformation

**Fig. 3:**
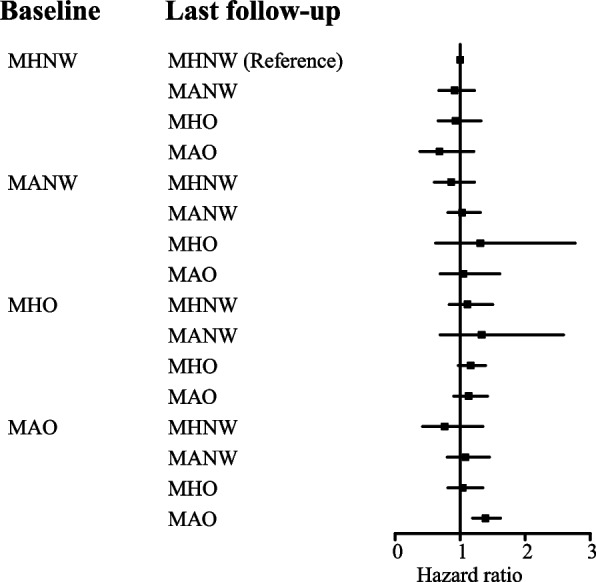
Risk for incident nephrolithiasis according to transition of metabolic overweight phenotypes

Furthermore, we investigated the effect of phenotypic changes in MAO subjects over time on the risk of developing new kidney stones. According to the phenotypic changes at the last follow-up, patients were divided into four groups. MAO subjects who transitioned to MHO (HR, 0.74; 95% CI, 0.58–0.95) or transitioned to MHNW (HR, 0.55; 95% CI, 0.31–0.98) were at a significantly decreased risk of incident nephrolithiasis, compared to MAO subjects who retained their phenotypes (Table 3). Besides, we studied the relationship between phenotypic changes in all subjects over time and risk of nephrolithiasis (Fig. 3). The results showed that the maintenance of MAO carried highest risk of incident kidney stones than persistent MHNW.

**Table 3:** Hazard ratios for incident kidney stone according to maintenance or transition of phenotypes in MAO group

Persistent subtypes (*n* = 6,997)		MAO at baseline			
		MHNW at last F/U	MHO at last F/U	MANW at last F/U	MAO at last F/U
Patient-years		403.23	1,830.28	1,242.83	7,560.94
Incidence rate per 1,000 patients-years (number of cases)		2.98(12)	4.04(74)	4.10(51)	5.30(401)
Crude HR^a^	HR (95% CI)	0.53(0.30–0.95)	0.77(0.60–0.98)	0.75(0.56–1.00)	1.00(reference)
	*P*-value	0.032	0.034	0.048	
Adjusted HR^b^	HR (95% CI)	0.55(0.31–0.98)	0.74(0.58–0.95)	0.79(0.59–1.06)	1.00(reference)
	*P*-value	0.042	0.018	0.114	

^a^A crude analysis without adjustment

^b^Adjusted for age, sex

## Discussion

In this large prospective long-term (6-year) cohort study, we identified that the risk of nephrolithiasis was most pronounced in the MAO group, while MHO individuals were also exhibited a 1.19-fold increased risk of kidney stones. Notably, during follow-up period, the risk of incident kidney stones significantly decreased as MAO subjects transitioned to other phenotypes without MA. This underscores how metabolic disorders can enhance the importance of overweight in the development of nephrolithiasis.

Many epidemiologic studies have identified the association between overweight and kidney stones [17, 18]. A complex scenario has been proposed, wherein overweight fosters both lithogenic process and insulin resistance, while overweight and insulin resistance jointly promote various metabolic abnormalities, which, in turn, act as determinants of nephrolithiasis [18, 19]. Therefore, we evaluate the risk of nephrolithiasis among individuals cross-classified by metabolic overweight phenotypes, which may help illustrate the role of overweight in the development of renal stones. A specific subgroup of overweight phenotype, referred to MHO, exhibits relatively fewer accompanying MA, such as fewer rates of diabetes, and hypertension. We identified that the risk of incident nephrolithiasis was significantly higher in MHO individuals than those with MHNW and MANW. More interestingly, when we examined the relationship between metabolic overweight status and the risk of kidney stones stratified by age and gender, a significantly increased risk of nephrolithiasis in both MHO and MAO status was observed in men < 60 years old. They all validated the hypothesis that overweight can contribute to renal stones formation even in the absence of MA.

A cohort study by Kim et al. indicated that the hazard ratio of incident nephrolithiasis was 1.12 in the MHO group, while the risk in MAO was much higher than that in MHO [20]. However, no Chinese cohort studies have explored the relationship between metabolic overweight phenotypes and nephrolithiasis. Our study with approximately 10,000 Chinese participants showed that the MHO and MAO groups were associated with an increased risk of kidney stones, which was consistent with the above Korean study.

Furthermore, MA status could change over time [21], and people are concerned that the association between metabolic overweight subtypes and kidney stones can be confused by chronological changes. Given this background, we conducted in-depth analysis according to maintenance or transition of metabolic overweight phenotypes during follow-up. During follow-up, approximately 70% of MAO individuals retained the same phenotype at the last examination and the remaining 30% changed to other phenotypes. We found that the risk of nephrolithiasis incidence in MAO status was significantly reduced as long as their metabolic abnormalities was altered (changing to MHNW or MHO), suggesting that prevention of kidney stones should emphasize the importance of maintaining metabolic health regardless of body weight. Besides, we identified that a small number of participants (8.19%) changed from MHNW to MHO throughout the follow-up period. Approximately 26% of the participants with initial MHO converted to MAO, which is lower than the 41%-48% conversion rates in Western population over 8–12 years of follow-up [22, 23], possibly due to our shorter follow-up time than that of Westerners.

Several potential mechanisms might help to understand how obesity itself contributes the formation of kidney stones, even in individuals with metabolic health who are relatively sensitive to insulin. Obesity not only promotes chronic systemic inflammation and oxidative stress, leading to tissue immune cell infiltration and the formation of kidney stones, but also increases adipokine expression and changes the status of inflammatory molecules, including interleukin-6 and tumor necrosis factor-$\alpha$ [24, 25]. Besides, overweight and obese individuals may increase their total caloric intake or engage in a lithogenic diet, further leading to a higher risk of nephrolithiasis [26]. A rat model study found that a weight loss intervention could reduce the risk of nephrolithiasis, supporting above findings [27]. In our study, although the association between persistent MHO and incident nephrolithiasis was marginally positive, the unstable nature of MHO subtypes may contribute to the development of incident nephrolithiasis. Thus, it is important to distinguish MHO from MHNW and MAO individuals.

Several limitations of this study require consideration. Firstly, our study used BMI as a marker for defining overweight, which cannot provide accurate measurements of adiposity, nor can it distinguish between muscle and fat, visceral and subcutaneous fat, or peripheral and central fat. Although waist circumference could help to rule out the possibility that some overweight patients had increased insulin resistance without MA, this examination was not included in our routine health check until January 2021. Therefore, metabolically healthy participants with isolated insulin resistance or visceral adiposity could be misclassified. Secondly, since the BMI classifications in our study are used according to Chinese standard, which is lower than the WHO criteria [16], and we only use 24 kg/m^2^ threshold to definite overweight status, without defining the various stages of obesity (normal weight, overweight and obesity), these all make it difficult to make a thorough comparison with other studies [28]. Thirdly, we are unable to account for dietary information that may have affected both adiposity level and nephrolithiasis. In addition, information on hospitalization or specific medication that could have affected nephrolithiasis was not available. Despite these limitations, our study boasts important strengths. This was a population-based cohort study with a large sample size and long-term observation period. In particular, tracking changes in metabolic overweight phenotypes during follow-up makes our findings more robust than other studies.

## Conclusions

In conclusion, our study identified that overweight, even without metabolic abnormalities, is still a vital risk factor for kidney stones formation independent of these common metabolic disorders in Chinese adults. Importantly, this work supports the notion that individuals with MAO should strive to achieve metabolic health status regardless of their weight, with the aim to reduce incidence of nephrolithiasis in the future.

### Supplementary Information

Supplementary Material 1.

## Data Availability

No datasets were generated or analysed during the current study.
