# Supplementary material for: ﻿Changes in metabolic overweight phenotypes over time and risk of nephrolithiasis: a cohort study
Source: BMC Public Health. 2024 Jul 16;24:1898. doi: 10.1186/s12889-024-19229-8 (PMC11251252; doi:10.1186/s12889-024-19229-8)

Supplementary Table 1. Baseline characteristics of participants among status of nephrolithiasis.

| Variables ^b^ | Nephrolithiasis | | P value ^a^ |
| --- | --- | --- | --- |
|  | No(n=8,847) | Yes(n=1,468) |  |
| Follow-up duration (years) | 4.13(2.04-5.02) | 3.02(1.73-4.01) | < 0.001 |
| Age | 57 (45, 67) | 58 (50, 67) | 0.003 |
| Male (n, %) | 5265(59.51) | 1061(72.28) | < 0.001 |
| BMI (kg/m^2^) | 24.1 (22.1, 26.3) | 24.8 (22.8, 26.9) | < 0.001 |
| Systolic pressure (mmHg) | 130 (117, 144) | 133 (121, 146) | < 0.001 |
| Diastolic pressure (mmHg) | 77 (70, 84) | 79 (72, 86) | < 0.001 |
| Glucose (mmol/L) | 5.58 (5.2, 6.09) | 5.69 (5.28, 6.25) | < 0.001 |
| Total cholesterol (mmol/L) | 4.98 (4.36, 5.63) | 5.04 (4.43, 5.72) | 0.01 |
| Triglyceride (mmol/L) | 1.27 (0.92, 1.8) | 1.39 (1, 1.96) | < 0.001 |
| HDL (mmol/L) | 1.32 (1.13, 1.56) | 1.28 (1.11, 1.5) | < 0.001 |
| LDL (mmol/L) | 3.02 (2.48, 3.6) | 3.1 (2.51, 3.68) | 0.008 |
| Hypertension (n, %) |  |  | < 0.001 |
| No | 3829(43.28) | 518(35.29) |  |
| Yes | 5018(56.72) | 950(64.71) |  |
| Diabetes (n, %) |  |  | 0.001 |
| No | 6579(74.36) | 1033(70.37) |  |
| Yes | 2268(25.64) | 435(29.63) |  |
| Baseline phenotypes |  |  | < 0.001 |
| MHNW | 3159(35.71) | 408(27.79) |  |
| MHO | 2077(23.48) | 366(24.93) |  |
| MANW | 1062(12) | 156(10.63) |  |
| MAO | 2549(28.81) | 538(36.65) |  |

^a^ Comparisons between groups analyzed by ANOVA or Kruskal-Wallis test for continuous variables; and Chi-squared test was used to examine the differences for categorical variables;

^b^ MHNW = metabolically healthy normal weight; MHO =﻿ metabolically healthy overweight; MANW = metabolically abnormal normal weight; MAO = metabolically abnormal overweight; BMI = body mass index; HDL-C=high density lipoprotein cholesterol; LDL-C = low-density lipoprotein cholesterol.

Supplementary Table 2. Hazard ratios for kidney stone according to presence of obesity and metabolic abnormality

| Baseline phenotypes (n=10,315) | | Normal | Overweight | Without MA | With MA |
| --- | --- | --- | --- | --- | --- |
| Patient-years | | 17,269 | 19,651 | 21,420 | 15,501 |
| Incidence rate per 1,000 patients-years (number of cases) | | 3.27(564) | 4.60(904) | 3.61(774) | 4.48(694) |
| Crude HR ^a^ | HR (95% CI) | 1.00(reference) | 1.43(1.28-1.59) | 1.00(reference) | 1.23(1.11-1.37) |
|  | P-value |  | <0.001 |  | <0.001 |
| Adjusted HR ^b^ | HR (95% CI) | 1.00(reference) | 1.25(1.12-1.39) | 1.00(reference) | 1.14(1.03-1.27) |
|  | P-value |  | <0.001 |  | 0.014 |

^a^ A crude analysis without adjustment;

^b^ Adjusted for age, sex.

Supplementary Table 3. Hazard ratios for kidney stone stratified by age and gender.

| **Gender** | **Age Groups** | **OR (95%CI)** | **P value** |
| --- | --- | --- | --- |
|  | Age ≥ 60 years old |  |  |
| Man | MHNW | Ref |  |
|  | MANW | 0.98(0.73-1.33) | 0.907 |
|  | MHO | 1.00(0.76-1.30) | 0.982 |
|  | MAO | 1.08(0.85-1.36) | 0.535 |
| Woman | MHNW | Ref |  |
|  | MANW | 0.99(0.66-1.49) | 0.958 |
|  | MHO | 1.07(0.70-1.65) | 0.744 |
|  | MAO | 1.34(0.94-1.91) | 0.102 |
|  | Age < 60 years old |  |  |
| Man | MHNW | Ref |  |
|  | MANW | 1.00(0.68-1.48) | 0.991 |
|  | MHO | 1.40(1.11-1.76) | 0.005 |
|  | MAO | 1.49(1.19-1.87) | 0.001 |
| Woman | MHNW | Ref |  |
|  | MANW | 1.21(0.73-2.00) | 0.466 |
|  | MHO | 0.95(0.65-1.40) | 0.805 |
|  | MAO | 1.64(1.13-2.37) | 0.009 |

Supplementary Table 4. Baseline characteristics of participants among 4 persistent phenotypes classified by the presence overweight and/or metabolic abnormality

| **Variables** ^b^ | **Overall** | **Normal weight** | |  | **Overweight** | | **P value ^a^** |
| --- | --- | --- | --- | --- | --- | --- | --- |
|  |  | **MHNW** | **MANW** |  | **MHO** | **MAO** |  |
| **Participants (n, %)** | 6,977 | 2,735(39.09%) | 704(10.06%) |  | 1411(20.17%) | 2147(30.68%) | - |
| **Follow-up duration (years)** | 4.00(1.98,5.00) | 4.00(1.99,5.00) | 4.13(1.98,5.02) |  | 3.97(1.97,4.95) | 4.02(1.96,5.01) | < 0.001 |
| **Age** | 56 (45, 67) | 52 (37, 63) | 66 (56, 76) |  | 54 (42, 64) | 61 (52, 70) | < 0.001 |
| **Male (n, %)** | 4171 (60) | 1099 (40.2%) | 386 (54.8%) |  | 1048 (74.3%) | 1638 (76.3%) | < 0.001 |
| **BMI (kg/m^2^)** | 24.1 (21.7, 26.6) | 21.4 (20, 22.5) | 22.4 (21.4, 23.2) |  | 25.9 (24.9, 27.4) | 27 (25.7, 28.7) | < 0.001 |
| **Systolic pressure (mmHg)** | 130 (117, 144) | 118 (109, 129) | 142 (133, 155) |  | 127 (117, 139) | 140 (131, 153) | < 0.001 |
| **Diastolic pressure (mmHg)** | 77 (70, 84) | 72 (66, 78) | 79 (72, 86) |  | 77 (71, 83) | 83 (76, 90) | < 0.001 |
| **Glucose (mmol/L)** | 5.57 (5.18, 6.08) | 5.29 (4.99, 5.62) | 6.26 (5.6, 7.22) |  | 5.42 (5.12, 5.74) | 6.16 (5.57, 6.99) | < 0.001 |
| **Total cholesterol (mmol/L)** | 4.97 (4.36, 5.61) | 4.96 (4.35, 5.55) | 5.02 (4.34, 5.7) |  | 4.93 (4.42, 5.55) | 4.99 (4.35, 5.74) | 0.078 |
| **Triglyceride (mmol/L)** | 1.24 (0.89, 1.82) | 0.93 (0.72, 1.19) | 1.75 (1.18, 2.3) |  | 1.13 (0.91, 1.41) | 1.99 (1.5, 2.68) | < 0.001 |
| **HDL (mmol/L)** | 1.33 (1.13, 1.59) | 1.57 (1.36, 1.78) | 1.21 (1.05, 1.4) |  | 1.36 (1.2, 1.54) | 1.1 (0.98, 1.26) | < 0.001 |
| **LDL (mmol/L)** | 2.99 (2.45, 3.57) | 2.91 (2.41, 3.46) | 3 (2.4, 3.65) |  | 3.11 (2.65, 3.61) | 3.01 (2.41, 3.65) | < 0.001 |
| **Hypertension (n, %)** |  |  |  |  |  |  | < 0.001 |
| **No** | 3062 (44) | 1977 (72.3%) | 85 (12.1%) |  | 759 (53.8%) | 241 (11.2%) |  |
| **Yes** | 3935 (56) | 758 (27.7%) | 619 (87.9%) |  | 652 (46.2%) | 1906 (88.8%) |  |
| **Diabetes (n, %)** |  |  |  |  |  |  | < 0.001 |
| **No** | 5232 (75) | 2637 (96.4%) | 272 (38.6%) |  | 1338 (94.8%) | 985 (45.9%) |  |
| **Yes** | 1765 (25) | 98 (3.6%) | 432 (61.4%) |  | 73 (5.2%) | 1162 (54.1%) |  |
| **Nephrolithiasis (n, %)** |  |  |  |  |  |  | < 0.001 |
| **No** | 5987 (86) | 2425 (88.7%) | 612 (86.9%) |  | 1204 (85.3%) | 1746 (81.3%) |  |
| **Yes** | 1010 (14) | 310 (11.3%) | 92 (13.1%) |  | 207 (14.7%) | 401 (18.7%) |  |

^a^ Comparisons between groups analyzed by ANOVA or Kruskal-Wallis test for continuous variables; and Chi-squared test was used to examine the differences for categorical variables;

^b^ MHNW = ﻿metabolically healthy normal weight; MHO =﻿ metabolically healthy overweight; MANW = metabolically abnormal normal weight; MAO = metabolically abnormal overweight; BMI = body mass index; HDL-C = high density lipoprotein cholesterol; LDL-C = low-density lipoprotein cholesterol.

Supplementary Table 5. Hazard ratios for kidney stone among 4 persistent phenotypes classified by the presence of overweight and/or metabolic abnormality

| **Persistent subtypes (n=6,997)** | | **MHNW** | **MANW** | **MHO** | **MAO** |
| --- | --- | --- | --- | --- | --- |
| Patient-years | | 9,651.29 | 2,569.56 | 4,836.94 | 7,560.94 |
| Incidence rate per 1,000 patients-years (number of cases) | | 3.21(310) | 3.58(92) | 4.28(207) | 5.30(401) |
| Crude HR ^a^ | HR (95% CI) | 1.00(reference) | 1.10(0.87-1.39) | 1.37(1.15-1.63) | 1.65(1.43-1.92) |
|  | P-value |  | 0.417 | <0.001 | <0.001 |
| Adjusted HR ^b^ | HR (95% CI) | 1.00(reference) | 1.06(0.83-1.34) | 1.17(0.98-1.41) | 1.42(1.21-1.66) |
|  | P-value |  | 0.656 | 0.084 | <0.001 |

^a^ A crude analysis without adjustment;

^b^ Adjusted for age, sex.

Supplementary Table 6. Hazard ratios for kidney stone among 4 different phenotypes classified by the presence of overweight and/or metabolic abnormality after excluding those newly developed nephrolithiasis

| **Persistent subtypes (n=9,962)** | | **MHNW** | **MANW** | **MHO** | **MAO** |
| --- | --- | --- | --- | --- | --- |
| Patient-years | | 12,716.48 | 4,424.92 | 8,531.23 | 10,899.33 |
| Incidence rate per 1,000 patients-years (number of cases) | | 2.49(317) | 2.64(117) | 3.31(282) | 3.66(399) |
| Crude HR ^a^ | HR (95% CI) | 1.00(reference) | 1.05(0.85-1.30) | 1.37(1.16-1.60) | 1.47(1.27-1.70) |
|  | P-value |  | 0.656 | <0.001 | <0.001 |
| Adjusted HR ^b^ | HR (95% CI) | 1.00(reference) | 0.99(0.80-1.23) | 1.21(1.03-1.43) | 1.27(1.09-1.49) |
|  | P-value |  | 0.913 | 0.023 | 0.002 |

^a^ A crude analysis without adjustment;

^b^ Adjusted for age, sex.

Supplementary Table 7. Prevalences of overweight/obese metabolic phenotypes at baseline and last follow-up visit

| **Baseline (N, %)** | | **Last follow-up (N, %)** | |
| --- | --- | --- | --- |
| **MHNW** | 3567(34.58%) | MHNW | 2735(76.68%) |
|  |  | MANW | 422(11.83%) |
|  |  | MHO | 292(8.19%) |
|  |  | MAO | 118(3.31%) |
| **MHO** | 2443(23.68%) | MHNW | 341(13.96%) |
|  |  | MANW | 51(2.09%) |
|  |  | MHO | 1411(57.76%) |
|  |  | MAO | 640(26.2%) |
| **MANW** | 1218(11.81%) | MHNW | 316(25.94%) |
|  |  | MANW | 704(57.8%) |
|  |  | MHO | 37(3.04%) |
|  |  | MAO | 161(13.22%) |
| **MAO** | 3087(29.93%) | MHNW | 109(3.53%) |
|  |  | MANW | 323(10.46%) |
|  |  | MHO | 508(16.46%) |
|  |  | MAO | 2147(69.55%) |

Supplementary Figure 1. Subgroup analyses. A comparison of the adjusted odds ratio of nephrolithiasis for the subgroups is presented by forest plot.


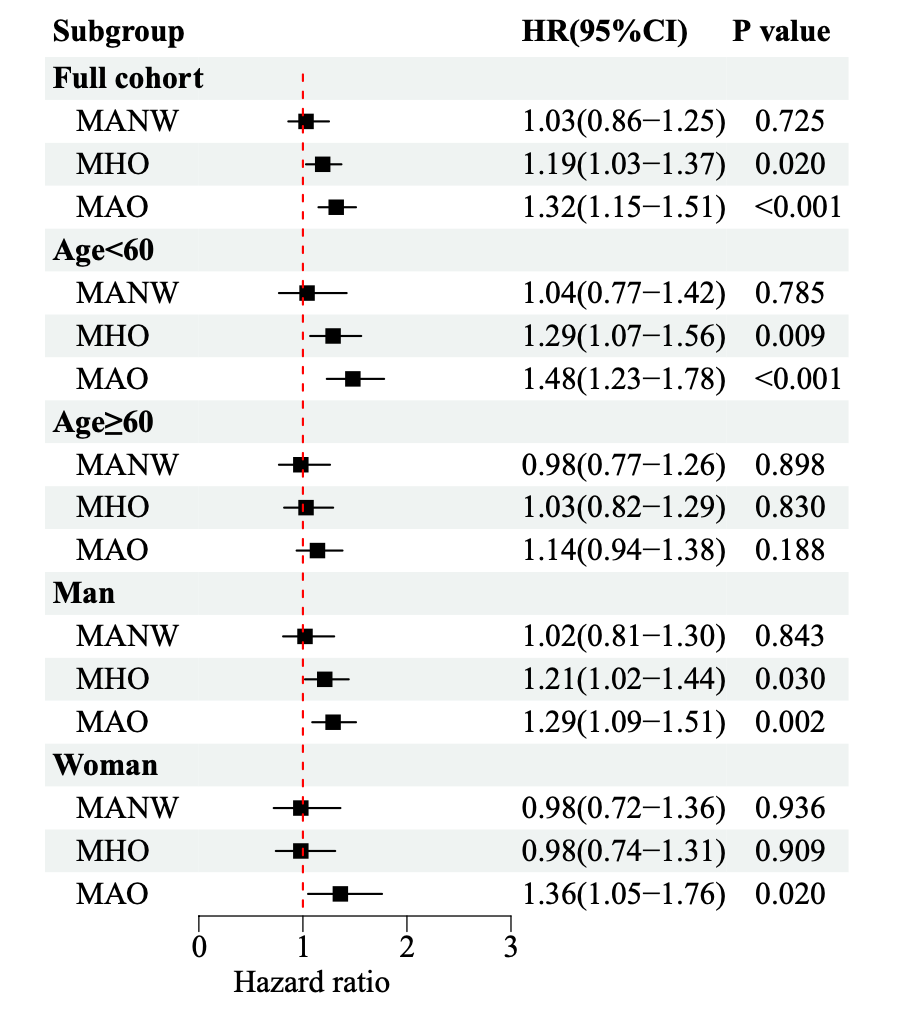

Supplement: Supplementary file 1 — Supplementary Material 1. [file 12889_2024_19229_MOESM1_ESM.docx]
